# The Move More Scholars Institute: A State Model of the Physical Activity and Public Health Practitioners Course

**Published:** 2007-06-15

**Authors:** Lori Schneider, Dianne Ward, Amber Vaughn, Carolyn Dunn, Jimmy Newkirk, Cathy Thomas

**Affiliations:** North Carolina Division of Public Health; University of North Carolina at Chapel Hill, Chapel Hill, NC; University of North Carolina at Chapel Hill, Chapel Hill, NC; North Carolina Cooperative Extension Service, North Carolina State University, Raleigh, NC; North Carolina Division of Public Health, Raleigh, NC; North Carolina Division of Public Health, Raleigh, NC

## Abstract

Physical activity has been identified as a public health priority. In response, training and professional development opportunities have been created to increase the capacity of public health practitioners to address this issue. Currently, training resources are primarily reaching national- and state-level professionals. Local-level physical activity and public health practitioners can also benefit from these resources. The Move More Scholars Institute, a 4-day training course for community-based physical activity practitioners in North Carolina, was developed for local practitioners. This article will describe the planning of, implementation of, and initial response to the Move More Scholars Institute.

## Background

Increasing physical activity among U.S. populations is a public health priority. Lack of physical activity has been associated with cardiovascular disease, hypertension, overweight, obesity, osteoporosis, diabetes, and certain cancers (1).

In 1996, the first U.S. Surgeon General's report on physical activity was released, highlighting the association between physical activity and health (2). Since the release of this report, increasing emphasis has been placed on physical activity. In 2000 physical activity was given emphasis for the first time in the *Dietary Guidelines for Americans *(3), and in 2005 the new *MyPyramid* continued to include physical activity recommendations (4). The *Guide to* *Community Preventive Services* for physical activity was released in 2001, containing systematic reviews of effective population-based physical activity interventions (5).

The increasing emphasis on the importance of physical activity coupled with the increasing evidence base for effective strategies to increase physical activity has created a need to translate what is known about effective interventions into the skills practitioners need to put the evidence into practice.

Since 1996, the Physical Activity and Public Health (PAPH) Course, which includes the Practitioner's Course on Community Interventions, has been offered annually by the Centers for Disease Control and Prevention (CDC) and the University of South Carolina Prevention Research Center to address this gap in public health expertise (6). Participants in the practitioner's course have been affiliated with colleges and universities, state departments of health, and many other organizations such as hospitals, local health departments, nonprofit organizations, private foundations, research institutes, and federal government agencies (6). However, participation is limited to 25 practitioners each year; the cost to attend (approximately $1300) also limits participation. As a result, this highly successful course, designed to address a critical gap, has limited impact on local physical activity practitioners.

Using the objectives of the PAPH Course as a starting point, the Physical Activity and Health Branch (PAHB)Using the objectives of the PAPH Course as a starting point, the Physical Activity and Health Branch (PAHB) in the Division of Nutrition and Physical Activity at CDC established five benchmarks, outlining the areas of training and technical assistance that state health departments need to address to improve their capacity to promote physical activity among the public: 1) develop and sustain effective partnerships; 2) use public health data as a tool to develop and prioritize community-based interventions; 3) understand and implement a sound approach to planning and evaluation; 4) implement evidence-based strategies at the informational, behavioral, and social and environmental policy levels; 5) and develop an organizational structure that contributes to program growth and sustainability by encouraging and supporting professional development and fostering successful collaborations within and outside the health department (7). Building on these benchmarks, a set of core competencies, specific to physical activity and public health practice, were developed by the National Society of Physical Activity Practitioners in Public Health (NSPAPPH) (8).

Building state and local infrastructure with the capacity to address the underlying causes of chronic disease is challenging (9), one of the challenges being to address the various educational backgrounds of community-based physical activity practitioners. Many of these individuals may have training in physical activity or public health but not adequate training in both. A well-trained public health workforce is essential if public health research is to have a tangible impact on populations (6). Using these benchmarks to create professional development opportunities that focus on the core competencies will increase the capacity of local physical activity and public health practitioners.

To increase the capacity of local community-based physical activity professionals and to overcome the barriers many may experience to attend the PAPH Course, the Physical Activity and Nutrition (PAN) Branch of the North Carolina Division of Public Health, in partnership with the University of North Carolina (UNC) at Chapel Hill, School of Public Health, developed a state-based version of the PAPH Course, called the Move More Scholars Institute (MMSI). This paper will describe the planning of, implementation of, and response to the MMSI course.

## Planning

The development of the MMSI was guided by an expert advisory committee made up of representatives from Active Living by Design, the PAPH Course, the North Carolina Division of Public Health, UNC at Chapel Hill, and the University of Tennessee at Chattanooga. The goal was to develop a course modeled after the PAPH Course, with a curriculum specific to North Carolina that addressed current and future trends of community-based physical activity initiatives. The resulting MMSI is an intensive 4-day training for community-based physical activity professionals in North Carolina. It is the first state-specific course modeled from the PAPH Course, integrating the core competencies established by the NSPAPPH. The inaugural MMSI was held in spring 2006. The PAN Branch plans to offer the MMSI annually, pending the availability of funding.

Objectives for the MMSI were to increase the capacity of community-based physical activity professionals to 1) use public health data and scientific information as tools to develop and prioritize community-based interventions; 2) use evidence-based and promising practice methods to implement community-based physical activity interventions and communicate physical activity messages; 3) understand the key components in a sound approach to evaluation; and 4) understand active living concepts.

The course was designed to support the statewide partnership effort in North Carolina called *Eat Smart, Move More…North Carolina *(10). This effort encourages people to eat healthfully and be physically active by creating policies, practices, and environments that promote and support these behaviors. The MMSI strengthens this effort through a curriculum focused on evidence-based interventions, including developing policies and environments to promote and support physical activity.

## Implementation

### Selection process

Marketing of the inaugural MMSI focused on three select groups working on community-based physical activity programming in North Carolina: 1) health promotion coordinators from local health departments, 2) coordinators of local physical activity and nutrition coalitions (LPANs), and 3) family and consumer science agents from cooperative extensions. These individuals were encouraged to share information about the course with any of their community partners who may have an interest in applying to attend. These professionals were targeted because they are leading the efforts in local communities to integrate physical activity interventions with an emphasis on policy- and environmental-level change. Eighty-five health promotion coordinators from local health departments and districts in North Carolina receive state and federal funding through the Statewide Health Promotion Program to implement policy and environmental change interventions that support physical activity, healthy eating, and tobacco use prevention and cessation. These coordinators often provide staff support to LPANs, which are coordinated by community partners and contribute to statewide programs encouraging physical activity and healthy eating. Family and consumer science agents throughout the state educate the public, influence public policy, and help families put research-based knowledge to work in their lives. Family and consumer science agents often partner with health promotion coordinators and are part of LPANs.

Professionals who attended the MMSI (referred to as "scholars") were chosen through an application process based on the selection process for the PAPH Course. Applicants were reviewed on the basis of their professional position in community-based physical activity programming, professional credentials, professional experience, support from their organizations, and the potential to impact the health of their counties by applying what they learned at the MMSI. A three-person review team consisting of two staff members from the North Carolina Division of Public Health and one from UNC at Chapel Hill reviewed all applications. Twenty-eight scholars attended the course held in Greensboro, NC. Twenty-three scholars were selected though the competitive application process and five state-level scholars were invited to attend on the basis of their ability to promote physical activity initiatives through their organizations. Scholars consisted of representatives from local health departments, local cooperative extensions, Recreation and Parks, North Carolina Division of Public Health, North Carolina Office on Disability and Health, North Carolina Academy of Family Physicians, and North Carolina State University Recreation Resource Services. MMSI scholars were from all areas of North Carolina.

### Funding

The MMSI was offered by the North Carolina Division of Public Health, PAN Branch. It was funded by Get Kids in Action*,* a partnership between UNC at Chapel Hill and The Gatorade Company that aims to increase physical activity among children to reduce and prevent obesity.

Scholarships were provided to all scholars, allowing them to attend at no cost to their organization. Most scholars were responsible only for travel costs to and from the course. Travel scholarships were made available to scholars traveling 300 miles or more in personal vehicles to attend the MMSI. This funding allowed 28 scholars to attend, with an average cost of approximately $1200 per participant.

### Course content

The course covered the planning, implementation, and evaluation of physical activity interventions with a North Carolina focus. The course began with a review of the physical activity recommendations for children and adults and the *Community Guide to Preventive Services for Physical Activity* and segued into practical application of the material. Agenda topics included understanding the physical activity recommendations for adults and children; understanding the *Community Guide to Preventive Services for Physical Activity;* using data in public health decision making; social marketing; key components to evaluation; understanding the policy process; designing healthy communities; and making partnerships work. Scholars were encouraged to learn from one another and were given time to debate and challenge one another with innovative ideas. (See Appendix for the complete agenda, including learning objectives.)

Each session of the MMSI was taught by nationally recognized faculty from Active Living by Design, CDC, the North Carolina Division of Public Health, and UNC at Chapel Hill. Informal networking took place during meals and between sessions, similar to networking during the PAPH Course. Formal networking was provided through faculty consultations, group work, and a group project.

### Response to the course

Scholars completed daily and overall course evaluations. Sessions were rated on a scale of poor, fair, good, very good, and excellent. The average rating of all of the sessions was very good, and the information was reported to be useful and applicable. Overall, scholars valued one-on-one time with faculty and gave positive feedback about the accommodations. When asked about the overall strengths of the MMSI, scholars reported valuing the in-depth information; the curriculum's successful integration of the problem and recommendations for effecting change; and the high-quality faculty assembled. Many scholars commented that the sessions on policy process and designing healthy communities were the most useful.

Scholars overwhelmingly reported that their knowledge of community-based physical activity interventions and public health had increased substantially as a result of attending the MMSI. They also agreed that participation in the MMSI would positively influence their future professional activities. Regarding weaknesses, the most common suggestion was to balance the agenda more between didactic and interactive sessions throughout the day.

No outcome data are available to evaluate the impact of the MMSI on the behavior of participants. However, an established monitoring system within the PAN Branch will be used to assess the MMSI success at one year. One strategy that has been implemented to facilitate communication among the scholar group is the use of a "wiki" (similar in design to Wikipedia) to encourage scholars to share activities among the group. It is too early to assess use and helpfulness of this tool.

### Lessons learned

Among the key lessons learned from the development and implementation of the MMSI are the following:

- Networking opportunities are maximized by limiting the number of participants.
- Participation of individuals with varied backgrounds enhances learning.
- Using qualified speakers with expertise on the state issues makes information immediately applicable for participants and enhances statewide efforts.
- Course content should be adapted to the needs of the participants.
- Venue is important: a quality environment contributes to the learning experience.

## Conclusion

The infrastructure to address physical activity varies by state, but the need to increase the capacity of local community-based practitioners to address physical activity as a public health priority is consistent throughout the country. A successful model has been created to address professional development needs of physical activity practitioners working in public health through the PAPH Course. Additionally, a core set of competencies has been developed by the NSPAPPH. Modifying the PAPH Course, integrating the core competencies, and incorporating state-specific content creates a professional development opportunity that is immediately relevant to the needs of local practitioners and that enhances statewide efforts to promote and support physical activity. Through the MMSI, North Carolina took steps to strengthen its local infrastructure by providing a professional development opportunity for local community-based physical activity professionals. The PAN Branch of the North Carolina Division of Public Health plans to seek funding to offer the course annually.

## Move More Scholars Institute Agenda

**Table:** Day 1

2:00 pm – 2:30 pm	Welcome/Introductions/Orientation
2:30 pm – 5:30 pm	Group Project Sharing Session Instructional Objective Each scholar will provide an overview of one community-based project they are working on that focuses on increasing physical activity for families and/or children
6:00 pm – 8:30 pm	Reception/Dinner

**Table:** Day 2

8:00 am – 9:00 am	Moving More: Understanding Physical Activity Recommendations for Children and Adults Session Instructional Objectives By the end of this session, scholars will be able to: Explain how the physical activity recommendations were developed Explain the physical activity recommendations for adults and children
9:00 am – 9:15 am	Break
9:15 am – 10:30 am	Obtaining Data in Your Community: What to Collect, Where to Collect it, and How to Use it Session Instructional Objectives By the end of this session, scholars will be able to: Determine when and how to use quantitative and qualitative data Identify existing sources of quantitative and qualitative data in your community Use data in public health decision making
10:30 am – 10:45 am	Break
10:45 am – 12:00 pm	An Introduction to the *Community Guide to Preventive Services*: Evidenced-Based Physical Activity Interventions Session Instructional Objectives By the end of this session, scholars will be able to: Define evidence-based and promising practice planning Describe uses and limitations of the *Community Guide* for physical activity interventions Translate effective intervention strategies, based on sound health education/evaluation strategies, to partners and other constituents
12:00 pm – 2:00 pm	Lunch/Walk/Faculty Consultations
2:00 pm – 3:30 pm	Community Change: An Integrated Approach to Helping NC Families Move More Session Instructional Objectives By the end of this session, scholars will be able to: Define the socioecological model Select appropriate informational, behavioral, social, environmental, and policy-level interventions to promote physical activity among North Carolina families Involve the efforts of worksites, coalitions, agencies, schools, and communities in attempts to change the local environment to create opportunities for physical activity
3:30 pm – 5:00 pm	Break/Faculty Consultations
5:00 pm – 6:00 pm	Dinner
6:00 pm – 7:15 pm	What Children Need to Move More: A Case Study to Increase Physical Activity Among NC Youth Session Instructional Objectives By the end of this session, scholars will be able to: Explain policy and environmental influences on physical activity levels among NC youth Understand the results from the FIT Together focus groups

**Table:** Day 3

8:00 am – 10:00 am	Simple Steps to Effective Evaluation Session Instructional Objectives By the end of this session, scholars will be able to: Explain the key components for evaluation of physical activity interventions focusing on environmental, policy, or direct behavior change Develop plans for evaluating physical activity interventions focusing on environmental, policy, or direct behavior change Write goals, SMART objectives, work plans, and evaluation measures for physical activity interventions
10:00 am – 10:15 am	Break
10:15 am – 12:00 pm	Designing Healthy Communities for Healthy Active Families Session Instructional Objectives By the end of this session, scholars will be able to: Understand the role of public health in designing healthy communities Apply a multi-level approach to designing healthy communities Explain the importance of multi-disciplinary partnerships for designing and supporting healthy communities
12:00 pm – 1:00 pm	Lunch
1:00 pm – 2:30 pm	Assessing Walkability: Seeing with New Eyes Session Instructional Objectives By the end of the session, scholars will be able to: Identify design concepts that influence the walkability of a community environment Learn to use a tool to audit the walkability of town and city streets Use the audit tool on streets near the conference center
2:30 pm – 3:00 pm	Break
3:00 pm – 4:30 pm	Promoting Health through the Policy Process: An IntroductionSession Instructional Objectives By the end of the session, scholars will be able to: List key concepts from theories of the policy process Describe how to use policy-process theories to coordinate a public health project meant to influence policy on a health issue
4:30 pm – 5:30 pm	Designing Healthy Communities for Healthy Active Families: Group Projects Session Instructional Objectives By the end of the session, scholars will be able to: Identify active advocates in their communities Apply a multilevel approach to designing healthy communities
5:30 pm – 6:30 pm	Dinner
6:30 pm – 7:30 pm	Designing Healthy Communities for Healthy Active Families: Group Projects Continued

**Table:** Day 4

8:00 am – 9:15 am	Designing Healthy Communities: Group Project Reports
9:15 am – 9:30 am	Break
9:30 am – 11:00 am	Making Partnerships Work for Health Promotion Session Instructional Objectives By the end of this session, scholars will be able to: Describe how partnerships facilitate population-based strategies for behavior change Identify their personality type and describe ways to work effectively with others Identify ways to leverage networks and collaborative partnerships
11:00 am – 11:30 am	Break/Check-out
11:30 am – 12:15 pm	The Future of Prevention for Obesity and Other Chronic Diseases: A National Perspective Session Instructional Objectives By the end of this session, scholars will be able to: Explain the future of the *Community Guide to Preventive Services* Understand the focus on policy and environmental changes to prevent obesity and other chronic diseases List CDC priorities to prevent obesity and other chronic diseases
12:15 pm – 12:30 pm	Scholars Slide Show/Closing Messages
12:30 pm	Lunch and Departure
